# Femtosecond Laser-Based Micromachining of Rotational-Symmetric Sapphire Workpieces

**DOI:** 10.3390/ma15186233

**Published:** 2022-09-08

**Authors:** Stefan Kefer, Julian Zettl, Cemal Esen, Ralf Hellmann

**Affiliations:** 1 Applied Laser and Photonics Group, Aschaffenburg University of Applied Sciences, Wuerzburger Strasse 45, 63743 Aschaffenburg, Germany; 2 Applied Laser Technologies, Ruhr-University Bochum, Universitaetsstrasse 150, 44801 Bochum, Germany

**Keywords:** sapphire, femtosecond laser, micromachining, laser turning, laser lathe, ablation threshold

## Abstract

Sapphire is a robust and wear-resistant material. However, efficient and high-quality micromachining is still a challenge. This contribution demonstrates and discusses two novels, previously unreported approaches for femtosecond laser-based micromachining of rotational-symmetric sapphire workpieces, whereas both methods are in principal hybrids of laser scanning and laser turning or laser lathe. The first process, a combination of a sequential linear hatch pattern in parallel to the workpiece’s main axis with a defined incremental workpiece rotation, enables the fabrication of sapphire fibers with diameters of 50 μm over a length of 4.5 mm. Furthermore, sapphire specimens with a diameter of 25 μm over a length of 2 mm can be fabricated whereas an arithmetical mean height, i.e., S_a_ parameter, of 281 nm is achieved. The second process combines a constant workpiece feed and orthogonal scanning with incremental workpiece rotation. With this approach, workpiece length limitations of the first process are overcome and sapphire fibers with an average diameter of 90 µm over a length of 20 cm are manufactured. Again, the sapphire specimen exhibits a comparable surface roughness with an average S_a_ value of 249 nm over 20 cm. Based on the obtained results, the proposed manufacturing method paves an innovative and flexible, all laser-based way towards the fabrication or microstructuring of sapphire optical devices, and thus, a promising alternative to chemical processes.

## 1. Introduction

Monocrystalline sapphire is one of the most durable materials since it features outstanding chemical, temperature, and corrosion resistance [1]. Therefore, it is nowadays used throughout a variety of scientific, industrial, and medical application fields. Typical examples are sapphire fibers for composite materials, sapphire substrates for optoelectronic devices and sensors, chemical ware, and optical windows or lenses [2,3]. On the other hand, due to its material properties, high-precision machining of sapphire remains a challenge. Thus, various scientific groups have been working on possible solutions for alternative sapphire machining approaches throughout recent years, whereas especially femtosecond laser-based processes are emerging as a promising method for efficient, high-quality, and high-precision machining of sapphire materials. In comparison to conventional methods, such as grinding, milling, and lapping, the non-contact processing approach via femtosecond lasers features minimized thermal effects [4,5,6,7]. While most contributions discuss surface ablation processes for bulk sapphire substrates [8,9,10,11,12,13,14,15,16,17,18], there are also publications with a focus on drilling [19,20,21], cutting [22,23], and the generation of photonic structures, such as integrated waveguides, Bragg gratings and polarizers [24,25,26,27,28,29]. Sapphire-based optical components are of great interest, since they enable long-term operation in high-temperature environments [30].

Machining of rotational-symmetric workpieces via laser turning with ultrashort pulse lasers is also a topic of constantly rising interest. In contrast to classical ablation or modification processes, this methodology is relatively novel and, thus, it is neither well explored nor is its potential fully exploited yet. However, recent studies already demonstrated promising results for the micromachining of metals as well as dielectrics by means of femtosecond laser turning [31,32,33,34]. Furthermore, Boerner et al. already conducted an initial study on laser turning of sapphire rods, reaching into the sub-mm range and, therefore, the micromachining regime. It is found that the realization of small feature sizes is still critical since unwanted heat accumulation effects lead to cracks and splinters on the machined specimen’s surface [35].

This contribution demonstrates two novel femtosecond laser-based approaches for micromachining of rotational-symmetric sapphire workpieces. Both are hybrid methods, combining laser scanning and turning processes. Depending on the employed method, sapphire fibers with outer diameters down to 25 µm, or with lengths up to 20 cm can be manufactured. The study also examines the resulting surface quality of both processes. Furthermore, the ablation threshold of monocrystalline sapphire for femtosecond pulses with different wavelengths is determined.

## 2. Materials and Methods

### 2.1. Sapphire Substrates and Fibers

The ablation threshold study is conducted on planar sapphire specimens (AL663025, Goodfellow, Hamburg, Germany). The substrates exhibit a quadratic footprint, with an edge length of 25 mm, and a thickness of 0.25 mm. Monocrystalline sapphire fibers (SF250-15 or SF150-50, Laser Components, Olching, Germany) are employed for the femtosecond laser-based microstructuring studies. The wrought material exhibits an outer diameter of 250 µm and a length of 15 cm, or a diameter of 150 µm and a length of 50 cm, respectively.

### 2.2. Laser Setup

**Figure 1:**
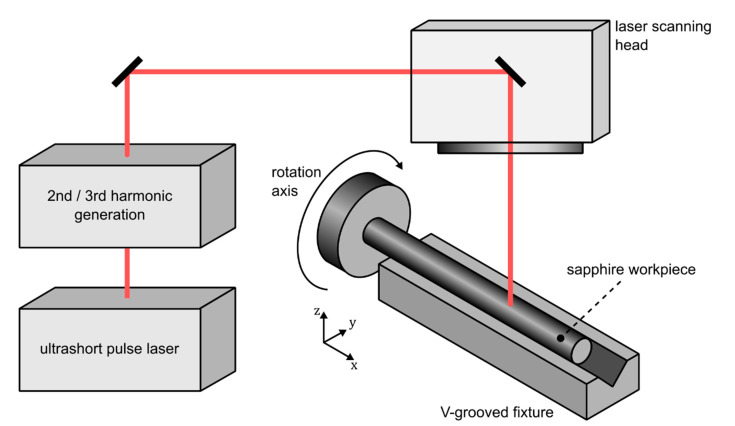
Schematic of the employed laser setup.

A schematic of the employed laser setup is depicted in Figure 1.

An ultrashort pulse laser system (Pharos-10-600 or Carbide, Light Conversion, Vilnius, Lithuania) featuring a 2nd and 3rd harmonic generation module is employed for all experiments discussed in this contribution. It enables irradiation of the samples with wavelengths of either 1030 nm, 515 nm, or 343 nm. Since it is equipped with a built-in pulse picker, the laser system is also able to emit a defined pulse quantity or single pulses, thus, enabling the single-shot laser damage threshold measurements. The radiation is imaged onto the sapphire workpieces by means of a laser scanning head (Miniscan II, Raylase, Wessling, Germany). While a focusing lens with a focal length of 100 mm is used for the ablation threshold experiments, a focal length of 55.6 mm is employed throughout all microstructuring studies. Although this leads to reduced focal spot diameters, the resulting Rayleigh range of 114 µm is still sufficient to omit the necessity of refocusing during the processes. Focal spot size quantification is realized by means of an adequate camera (UI-1490SE, IDS, Obersulm, Germany). While planar sapphire specimens are positioned on a conventional linear translation stage, cylindrical workpieces are mounted within a rotational axis (ACS-LP, Aerotech, Pittsburgh, PA, USA). Additionally, they are supported by a V-grooved fixture. The fixture consists of polyether ether ketone (PEEK), additionally supported by a stainless-steel mount. The employed V-groove is specifically tailored for this application with a depth of 400 µm and a bottom width of 200 µm, while its sidewalls exhibit an angle of 30 deg. With a length of 30 cm, the V-groove is capable of supporting workpieces up to an equal extent. All process parameters stated and discussed in Section 3 are determined empirically, with a focus on achieving the minimum fiber diameter over the maximum length.

### 2.3. Ablation Threshold Determination

A preliminary ablation threshold study is conducted in order to determine the optimum wavelength for the femtosecond laser-based microstructuring of sapphire. Therefore, the planar substrates are locally irradiated with different wavelengths. While pulse duration and repetition rate are kept constant with values of 230 fs and 100 kHz, each emission wavelength features a different focal spot diameter. For wavelengths of 1030 nm, 515 nm, and 343 nm, the respective focal spot sizes are determined experimentally as 36 µm, 21 µm, and 20 µm. By adapting the laser’s average output power, the resulting peak fluence within the focal spot is varied. The diameter of the induced ablation area is then evaluated via bright-field microscopy (DM6000M, Leica, Wetzlar, Germany). Theoretically, the squared diameter d^2^ of the ablation area correlates to the pulse peak fluence F according to

(1) $$d^{2}=2\omega_{0}^{2}\ln\left(\frac{F}{F_{\mathrm{th}}}\right),$$

whereas $\omega_{0}$ represents the focal spot radius while $F_{\mathrm{th}}$ is the ablation threshold fluence. Thus, it is possible to quantify the ablation threshold by plotting the squared diameter of the ablated areas as a function of the applied laser fluence in a semi-logarithmic manner. The ablation threshold is then determined by the respective linear least square fit function’s abscissa intersection [36,37].

### 2.4. Axial Scanning and Incremental Workpiece Rotation

**Figure 2:**
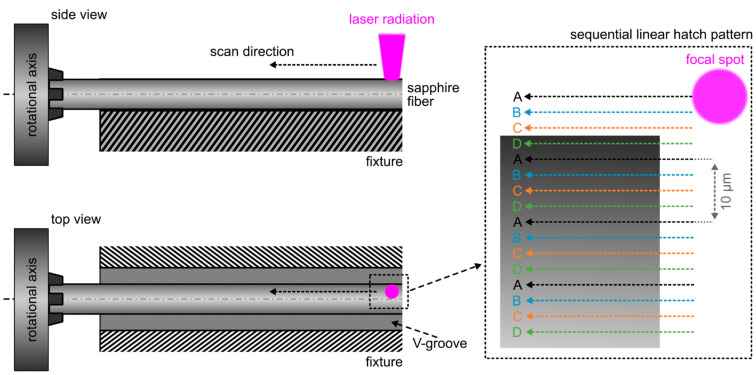
Femtosecond laser-based diameter reduction of sapphire rods by means of an axial scanning process combined with incremental workpiece rotation.

Monocrystalline sapphire fibers with an initial diameter of 250 µm and a length of 15 cm are fixed within the rotational mount on one side while most of the fiber rests within the V-grooved fixture. In this configuration, the resulting focal spot diameter is determined as 7 µm. Throughout the whole process, repetition rate, average output power, pulse duration, and pulse fluence are kept constant at 300 kHz, 1.11 W, 290 fs, and 9.6 J cm^−2^, respectively. The focal spot is positioned on the workpiece’s rotation axis, which prevents the necessity of in-situ refocusing. As outlined in Figure 2, a superposition of four individual linear hatch patterns (A, B, C, D) is used to remove material from the untreated sapphire specimen.

The scanning speed is chosen to obtain a resulting pulse overlap of approximately 65% along each line, whereas the lateral distance between two lines is 10 µm within each pattern. All hatch patterns are executed sequentially, starting with pattern A. Every subsequent hatch is offset laterally by 2.5 µm with respect to the previous one. This way, it is possible to maintain an effective lateral pulse overlap of 65% while, simultaneously, parasitic heat accumulation effects are drastically reduced. With 400 µm, the overall sequential hatch pattern width is chosen extensively larger than the initial fiber diameter to counter possible misalignments or process-related wobble movements.

After scanning of the whole superimposed hatch pattern, the sapphire fiber is rotated by an angle of 190 deg around the fiber’s optical axis with a speed of 200 deg·s^−1^. The rotation angle is deliberately chosen, since consistent material removal from both sides of the fiber is preferable to maintain its physical integrity. It is found that one-sided material removal as well as employing a rotation angle of 180 deg leads to instabilities during the process and results in bending or deformation of the sapphire fiber. The scanning process is repeated 36 times, which effectively yields an overall workpiece rotation of 360 deg. After this first iteration, the diameter of a 250 µm monocrystalline sapphire fiber is reduced to approximately 160 µm. Iterative repetition of the whole process finally defines the resulting fiber diameter.

### 2.5. Orthogonal Scanning Combined with Constant Workpiece Feed and Incremental Rotation

**Figure 3:**
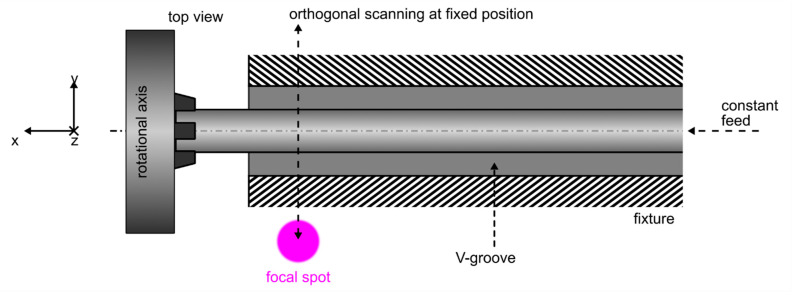
Femtosecond laser-based diameter reduction of sapphire rods over a length of 20 cm by means of an orthogonal scanning process.

Again, laser radiation with a wavelength of 343 nm is employed for this ablation process. While the pulse repetition rate is also 100 kHz, pulse duration, as well as spot diameter, are adapted to process-optimized values of 190 fs and 9.5 µm. The adapted average output power of 0.31 W yields a pulse fluence of 1.55 J cm^−2^. Throughout this procedure, the laser beam is scanned over the workpiece in an orthogonal direction to its major axis (*y*-direction) with a scanning speed of 400 mm·s^−1^. Simultaneously the specimen is translated alongside the *x*-axis at a constant feed rate of 0.45 mm·s^−1^. This parameter combination yields a pulse overlap of approximately 60% in *x*- as well as in *y*-direction. After a deliberately defined feed length, the workpiece is rotated by 210 deg before the ablation process starts over. Overall, this ablation procedure consists of 96 repetitions, which translates to 56 full rotations of the sapphire specimen. A schematic of the proposed microstructuring process is shown in Figure 3.

While the maximum feed length in the *x*-direction is only limited by the maximum translation range of the employed axis, the alignment precision of the supportive V-grooved fixture is insufficient for lengths beyond 1 cm. To counter unwanted misalignment effects by means of manual refocusing and repositioning, the sapphire workpiece is therefore processed in 1 cm sections. While this practically also results in the stitching of multiple working fields, no stitching errors are observed due to the orthogonal scanning approach. Again, the resulting diameter of the processed sapphire workpiece can be adapted by the number of process iterations.

## 3. Results and Discussion

### 3.1. Ablation Threshold of Sapphire

**Figure 4:**
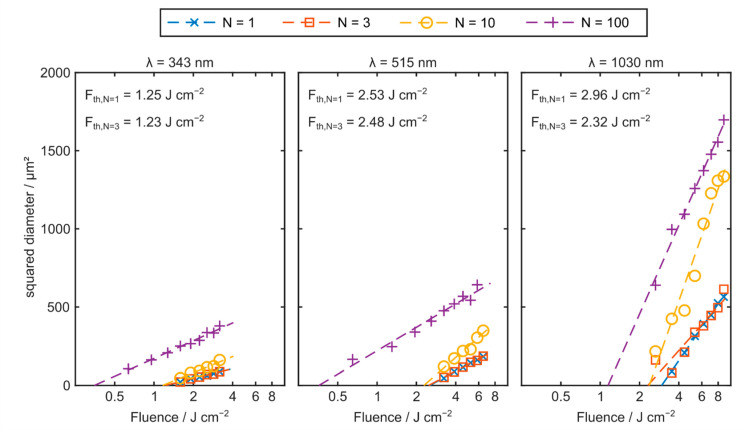
Squared diameter of the ablation areas as a function of the applied laser fluence for ultraviolet (λ = 343 nm), green (λ = 515 nm) and infrared (λ = 1030 nm) laser radiation. N is the pulse quantity. Each data point represents the average of three single measurements.

The determined squared ablation spot diameter as a function of the applied laser fluence is depicted in Figure 4. For a wavelength of 1030 nm, a single pulse ablation threshold fluence of 2.96 J cm^−2^ is observed. This value is comparable to previous ablation threshold studies utilizing femtosecond radiation in the infrared regime [8].

Furthermore, especially for pulse quantities of N ≤ 10, a significant reduction of the ablation threshold is found with decreasing wavelengths, whereas the single pulse ablation threshold for green and ultraviolet radiation is quantified as 2.53 and 1.25 J cm^−2^, respectively. In contrast, a deviating behavior is observed for larger pulse quantities (N = 100). While there is still a significant threshold reduction when green instead of infrared radiation is utilized, no further improvement is observed at a wavelength of 343 nm for this pulse quantity. This behavior is attributed to heat accumulation and microdamage [36,38]. However, especially in the outline of micromachining small-footprint workpieces, single- or few-pulse ablation is preferable to prevent suchlike effects. Consequently, based on this study, UV femtosecond radiation is generally preferable for the micromachining of sapphire workpieces.

### 3.2. Microstructuring via Axial Scanning and Incremental Workpiece Rotation

**Figure 5:**
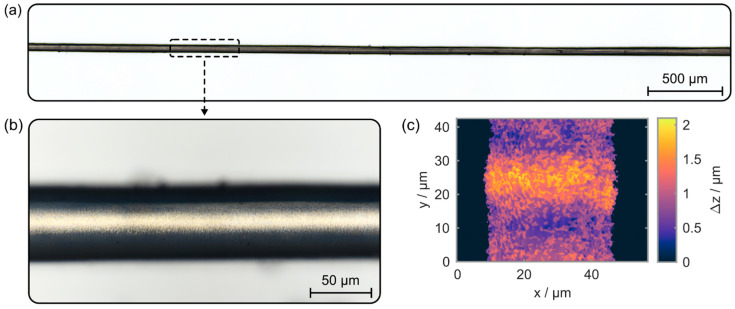
(**a**,**b**) Microscopic images of a sapphire fiber. It exhibits a reduced diameter of 50 µm over a length of 4.5 mm. (**c**) Surface roughness quantification via white-light interferometry.

Based on the proposed methodology, the diameter of sapphire fibers can be drastically reduced over an extended length, as shown in Figure 5.

Therein, exemplary microscopic images of a diameter-reduced sapphire fiber, manufactured by employing three iterations of the fabrication process, are depicted. With a diameter of 50 µm over a length of 4.5 mm, the specimen exhibits an aspect ratio of 90. After machining, the fiber is still straight and does not suffer from unwanted bending or deformations due to parasitic heat accumulation effects or intrinsic material tensions (see Figure 5a). Additionally, the specimen’s surface roughness is quantified with a white-light interferometer (WLI), whereas the height resolution of the employed device (Contour-GT, Bruker, Billerica, MA, USA) is specified as 0.1 nm by the manufacturer. Figure 5b,c depicts a high-resolution microscopic image as well as the sample’s height profile determined via WLI. Based on the determined mean surface area roughness, or S_a_ parameter, of 281 nm, the microstructured workpiece exhibits an outstanding surface quality. Except for a flattening algorithm to remove the fiber’s surface curvature, no additional filtering is employed in this evaluation process. Furthermore, no chipping, fissures, or cracks are evident on the machined specimen’s surface. The overall process time amounts to nine minutes which results in a diameter reduction speed of 22 µm·min^−1^. With length-related diameter reduction rates of up to 10 µm·cm·min^−1^, this femtosecond laser-based process is up to ten times faster than high-temperature wet etching approaches, currently, the only alternative to fabricate sapphire fibers with this dimension [39].

**Figure 6:**
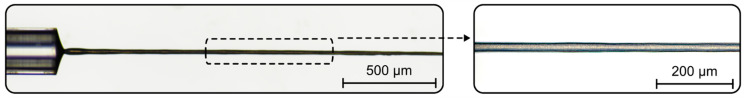
Microscopic images of a sapphire fiber with a reduced diameter of 25 µm.

As depicted in Figure 6, by executing a fourth iteration of the femtosecond laser-based diameter reduction process, the final sapphire fiber diameter can be further reduced to 25 µm.

Again, the final specimen is free from unwanted deformations. However, with this diameter, the maximum length is limited to 2 mm, which yields a maximum aspect ratio of 80, before thermal accumulation effects begin to compromise the structural integrity of the workpiece.

**Figure 7:**
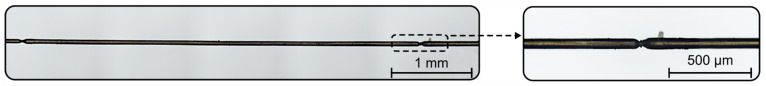
Microscopic images of stitching errors.

With this process, the maximum achievable length for diameter reductions down to 50 µm is defined by the effective scanning range of the applied combination of lateral translation stage and laser scanning head. Overall, the maximum scanning range is given as 5 mm. Thus, in order to achieve diameter reduction over extended lengths, stitching procedures are necessary. This, however, also results in stitching errors, as shown in Figure 7.

It is found that stitching multiple working fields next to each other leads to local neckings and, thus, predetermined breaking points. Stitching can, in general, be omitted by employing endless-feed systems, as already demonstrated in the outline of continuous metal fiber manufacturing or cutting machines based on endless diamond wires [40,41].

### 3.3. Microstructuring via Orthogonal Scanning Combined with Constant Workpiece Feed and Incremental Rotation

Alternatively, stitching errors can be avoided by employing this micromachining approach, which leads to a drastic increase in the achievable microstructuring length.

**Figure 8:**
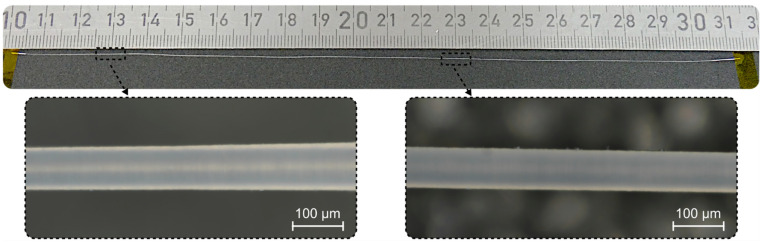
Sapphire fiber with an average outer diameter of 90 µm over a length of 20 cm, fabricated via orthogonal scanning in combination with incremental rotation of the workpiece and constant workpiece feed. Both zoom-ins show exemplary microscopic images at different positions along the fiber.

Figure 8 depicts a sapphire fiber whose outer diameter is reduced from 150 to 90 µm, over a length of 20 cm, by performing three iterations of the process. The resulting specimen exhibits a remarkable aspect ratio of 2200 while the overall length-related diameter reduction rate of this process is 0.6 µm·cm·min^−1^. However, at this point, it is worthwhile to note that both microstructuring methods discussed in this contribution are not yet optimized for process speed in any way.

**Figure 9:**
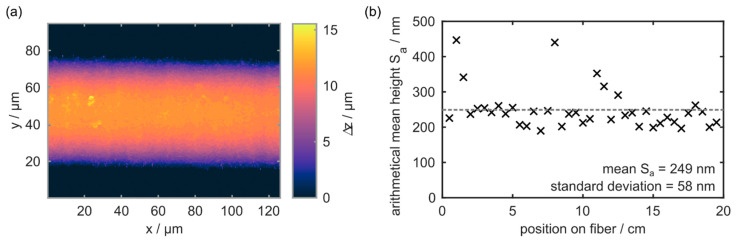
(**a**) Exemplary surface profile acquired via white-light interferometry. (**b**) Arithmetical mean height, or S_a_ value, of a micromachined sapphire fiber with an average diameter of 90 µm over a length of 20 cm.

The orthogonal scanning strategy also results in an outstanding surface quality, which is confirmed by white-light interferometry measurements. All results are acquired with a 200× objective and, except for a flattening algorithm to remove the fiber curvature, no additional filtering is applied during the evaluation of the raw data. The obtained S_a_ values along the fiber’s main axis, as well as an exemplary height profile acquired via WLI, are depicted in Figure 9.

On average, the arithmetical mean height along the sapphire workpiece is determined as 249 nm. While most S_a_ values are located in-between 200 and 300 nm, there are occasional outliers with S_a_ values up to 450 nm. This leads to a 1σ standard deviation of 58 nm. This also means that both femtosecond-laser based sapphire microstructuring strategies result in comparable surface qualities, which are in good agreement with surface roughness values resulting from femtosecond laser-based ablation processes on planar sapphire substrates [42].

## 4. Conclusions

In conclusion, this study demonstrates two novel femtosecond laser-based micromachining approaches for rotational symmetric sapphire workpieces. The first strategy is based on a sequential hatch scanning pattern with parallel orientation to the workpiece’s main axis, in combination with an incremental rotation process. With this approach, the diameter of sapphire rods can be reduced to 50 μm over a length of 4.5 mm, or 25 μm over a length of 2 mm. This results in aspect ratios of 90, or 80, respectively. The arithmetical mean height (S_a_ value) of the machined specimen is determined as 281 nm by means of white-light interferometry and the specimen’s surface is devoid of cracks, fissures, chip outs, and other unwanted defects. This contribution also discusses the current limitations of the proposed micromachining process based on axial scanning. Stitching errors, occurring at the overlap of multiple work areas, limit the maximum microstructuring length of this strategy. In contrast, the second approach, which is based on an orthogonal scanning process combined with a constant feed alongside the specimen’s main axis as well as incremental rotation of the workpiece, enables successful diameter reduction of sapphire rods down to 90 µm over a length of 20 cm. Thus, the final machined workpiece exhibits an aspect ratio of 2200. Furthermore, the specimen’s surface roughness is examined, resulting in an average S_a_ value of 249 nm, with a 1σ standard deviation of 58 nm over the whole workpiece length. The determined surface qualities of both processes are comparable to values achieved via femtosecond laser ablation of planar sapphire substrates. Consequently, both femtosecond laser-based micromachining approaches yield outstanding surface qualities while each process offers individual strengths in terms of achievable minimum diameter, maximum workpiece length, and process time. They additionally offer up to ten times increased fabrication speed in comparison with high-temperature wet-etching processes. Based on the achievable minimum diameters and the resulting surface quality, these newly developed machining processes pave the way toward novel manufacturing and/or microstructuring processes for sapphire-based optical fibers or sensors. Finally, this contribution also contains a comprehensive wavelength-dependent ablation threshold study for sapphire workpieces. It is found that, with an ablation threshold of 1.25 J cm^−2^, UV radiation is preferable for the micromachining of sapphire workpieces.

## Data Availability

Data underlying the results presented in this paper are not publicly available at this time but may be obtained from the authors upon reasonable request.

## References

[1] Harris D.C. (1999). Materials for Infrared Windows and Domes: Properties and Performance.

[2] Akselrod M.S., Bruni F.J. (2012). Modern trends in crystal growth and new applications of sapphire. J. Cryst. Growth.

[3] Pishchik V., Lytvynov L.A., Dobrovinskaya E.R. (2009). Sapphire.

[4] Pawar P., Ballav R., Kumar A. (2017). Machining Processes of Sapphire: An Overview. Int. J. Mod. Manuf. Technol..

[5] Maas P., Mizumoto Y., Kakinuma Y., Min S. (2017). Machinability study of single-crystal sapphire in a ball-end milling process. Int. J. Precis. Eng. Manuf..

[6] Mishra S., Yadava V. (2015). Laser Beam MicroMachining (LBMM)—A review. Opt. Lasers Eng..

[7] Bliedtner J., Schindler C., Seiler M., Wächter S., Friedrich M., Giesecke J. (2016). Ultrashort Pulse Laser Material Processing. Laser Tech. J..

[8] Eberle G., Schmidt M., Pude F., Wegener K. (2016). Laser surface and subsurface modification of sapphire using femtosecond pulses. Appl. Surf. Sci..

[9] Liu T., Wei H., Wu J., Lu J., Zhang Y. (2021). Modulation of crack formation inside single-crystal sapphire using ultrafast laser Bessel beams. Opt. Laser Technol..

[10] Qi L., Nishii K., Yasui M., Aoki H., Namba Y. (2010). Femtosecond laser ablation of sapphire on different crystallographic facet planes by single and multiple laser pulses irradiation. Opt. Lasers Eng..

[11] Shamir A., Ishaaya A.A. (2013). Large volume ablation of Sapphire with ultra-short laser pulses. Appl. Surf. Sci..

[12] Yin K., Duan J., Sun X., Wang C., Luo Z. (2015). Formation of superwetting surface with line-patterned nanostructure on sapphire induced by femtosecond laser. Appl. Phys. A.

[13] Varel H., Wähmer M., Rosenfeld A., Ashkenasi D., Campbell E. (1998). Femtosecond laser ablation of sapphire: Time-of-flight analysis of ablation plume. Appl. Surf. Sci..

[14] Li X., Jia T., Feng D., Xu Z. (2004). Ablation induced by femtosecond laser in sapphire. Appl. Surf. Sci..

[15] Liu H., Li Y., Lin W., Hong M. (2020). High-aspect-ratio crack-free microstructures fabrication on sapphire by femtosecond laser ablation. Opt. Laser Technol..

[16] Elgohary A., Block E., Squier J., Koneshloo M., Shaha R.K., Frick C., Oakey J., Aryana S.A. (2020). Fabrication of sealed sapphire microfluidic devices using femtosecond laser micromachining. Appl. Opt..

[17] Schwarz S., Rung S., Esen C., Hellmann R. (2018). Homogeneous Low Spatial Frequency LIPSS on Dielectric Materials Generated by Beam-Shaped Femtosecond Pulsed Laser Irradiation. J. Laser Micro/Nanoeng..

[18] Cai X., Ji C., Li C., Tian Z., Wang X., Lei C., Liu S. (2021). Multiphoton Absorption Simulation of Sapphire Substrate under the Action of Femtosecond Laser for Larger Density of Pattern-Related Process Windows. Micromachines.

[19] Lott G., Falletto N., Devilder P.-J., Kling R. (2016). Optimizing the processing of sapphire with ultrashort laser pulses. J. Laser Appl..

[20] Jo H., Ito Y., Hattori J., Nagato K., Sugita N. (2021). High-speed observation of damage generation during ultrashort pulse laser drilling of sapphire. Opt. Commun..

[21] Kuriakose A., Bollani M., Di Trapani P., Jedrkiewicz O. (2022). Study of Through-Hole Micro-Drilling in Sapphire by Means of Pulsed Bessel Beams. Micromachines.

[22] Xiao H., Zhang W., Zhou Y., Liu M., Zhou G. (2022). A Numerical Simulation and Experimental Study on the Ultrafast Double-Laser Precision Cutting of Sapphire Materials. Crystals.

[23] Tokarev V.N., Melnikov I.V. (2021). A Strategy for Achieving Smooth Filamentation Cutting of Transparent Materials with Ultrafast Lasers. Appl. Sci..

[24] Bérubé J.-P., Lapointe J., Dupont A., Bernier M., Vallée R. (2019). Femtosecond laser inscription of depressed cladding single-mode mid-infrared waveguides in sapphire. Opt. Lett..

[25] Kefer S., Roth G.-L., Zettl J., Schmauss B., Hellmann R. (2022). Sapphire Photonic Crystal Waveguides with Integrated Bragg Grating Structure. Photonics.

[26] Xu X., He J., He J., Xu B., Chen R., Wang Y., Yang Y., Wang Y. (2021). Efficient point-by-point Bragg grating inscription in sapphire fiber using femtosecond laser filaments. Opt. Lett..

[27] Guo Q., Yu Y.-S., Zheng Z.-M., Chen C., Wang P.-L., Tian Z.-N., Zhao Y., Ming X.-Y., Chen Q.-D., Yang H. (2019). Femtosecond Laser Inscribed Sapphire Fiber Bragg Grating for High Temperature and Strain Sensing. IEEE Trans. Nanotechnol..

[28] Wang M., Salter P.S., Payne F.P., Shipley A., Morris S.M., Booth M.J., Fells J.A.J. (2022). Single-mode sapphire fiber Bragg grating. Opt. Express.

[29] Fan H., Ryu M., Honda R., Morikawa J., Li Z.-Z., Wang L., Maksimovic J., Juodkazis S., Chen Q.-D., Sun H.-B. (2019). Laser-Inscribed Stress-Induced Birefringence of Sapphire. Nanomaterials.

[30] Yang S., Homa D., Heyl H., Theis L., Beach J., Dudding B., Acord G., Taylor D., Pickrell G., Wang A. (2019). Application of Sapphire-Fiber-Bragg-Grating-Based Multi-Point Temperature Sensor in Boilers at a Commercial Power Plant. Sensors.

[31] Warhanek M., Walter C., Hirschi M., Boos J., Bucourt J.F., Wegener K. (2016). Comparative analysis of tangentially laser-processed fluted polycrystalline diamond drilling tools. J. Manuf. Processes.

[32] Zettl J., Klar M., Rung S., Esen C., Hellmann R. (2021). Laser turning with ultrashort laser pulses. J. Manuf. Processes.

[33] Zettl J., Rung S., Esen C., Hellmann R. (2021). Tangential Laser Turning of Fused Silica Using Ultra-short Laser Pulses. J. Laser Micro/Nanoeng..

[34] Zettl J., Klar M., Esen C., Hellmann R. (2020). Generation of Rotationally Symmetric Micro Tools using Ultrashort Laser Pulses. J. Laser Micro/Nanoeng..

[35] Boerner P., Hajri M., Wahl T., Weixler J., Wegener K. (2019). Picosecond pulsed laser ablation of dielectric rods: Angle-dependent ablation process model for laser micromachining. J. Appl. Phys..

[36] Wang X.C., Lim G.C., Zheng H.Y., Ng F.L., Liu W., Chua S.J. (2004). Femtosecond pulse laser ablation of sapphire in ambient air. Appl. Surf. Sci..

[37] Liu J.M. (1982). Simple technique for measurements of pulsed Gaussian-beam spot sizes. Opt. Lett..

[38] Taylor L.L., Qiao J., Qiao J. (2016). Optimization of femtosecond laser processing of silicon via numerical modeling. Opt. Mater. Express.

[39] Hill C., Homa D., Yu Z., Cheng Y., Liu B., Wang A., Pickrell G. (2017). Single Mode Air-Clad Single Crystal Sapphire Optical Fiber. Appl. Sci..

[40] Teicher U., Lange T., Ihlenfeldt S. (2021). High performance machining of continuous metal fibers with cascaded multi-stage profile tools. Procedia CIRP.

[41] Qiu J., Liu C., Zhang S. (2021). The machining accuracy and surface roughness of mono-crystalline silicon regarding wire lag and wire stiffness of endless diamond wire. Int. J. Adv. Manuf. Technol..

[42] Pallarés-Aldeiturriaga D., Claudel P., Granier J., Travers J., Guillermin L., Flaissier M.-O., d’Augeres P.B., Sedao X. (2021). Femtosecond Laser Engraving of Deep Patterns in Steel and Sapphire. Micromachines.

